# Deletion of Glyoxalase 1 Exacerbates Acetaminophen-Induced Hepatotoxicity in Mice

**DOI:** 10.3390/antiox13060648

**Published:** 2024-05-25

**Authors:** Prakashkumar Dobariya, Wei Xie, Swetha Pavani Rao, Jiashu Xie, Davis M. Seelig, Robert Vince, Michael K. Lee, Swati S. More

**Affiliations:** 1 Center for Drug Design, College of Pharmacy, University of Minnesota, Minneapolis, MN 55455, USA; dobar004@umn.edu (P.D.); wei.xie100@gmail.com (W.X.); rao00122@umn.edu (S.P.R.); jxie@umn.edu (J.X.); vince001@umn.edu (R.V.); 2 Comparative Pathology Shared Resource, Masonic Cancer Center, University of Minnesota, St. Paul, MN 55108, USA; dseelig@umn.edu; 3 College of Veterinary Medicine, University of Minnesota, St. Paul, MN 55108, USA; 4 Department of Neuroscience, University of Minnesota, Minneapolis, MN 55455, USA; mklee@umn.edu; 5 Institute for Translational Neuroscience, University of Minnesota, Minneapolis, MN 55455, USA

**Keywords:** acetaminophen, glyoxalase 1, ψ-glutathione, hepatotoxicity, methylglyoxal, advanced glycation end product, antidote

## Abstract

Acetaminophen (APAP) overdose triggers a cascade of intracellular oxidative stress events, culminating in acute liver injury. The clinically used antidote, N-acetylcysteine (NAC), has a narrow therapeutic window, and early treatment is essential for a satisfactory therapeutic outcome. For more versatile therapies that can be effective even at late presentation, the intricacies of APAP-induced hepatotoxicity must be better understood. Accumulation of advanced glycation end products (AGEs) and the consequent activation of the receptor for AGEs (RAGE) are considered one of the key mechanistic features of APAP toxicity. Glyoxalase 1 (Glo-1) regulates AGE formation by limiting the levels of methylglyoxal (MEG). In this study, we studied the relevance of Glo-1 in the APAP-mediated activation of RAGE and downstream cell death cascades. Constitutive Glo-1-knockout mice (GKO) and a cofactor of Glo-1, ψ-GSH, were used as tools. Our findings showed elevated oxidative stress resulting from the activation of RAGE and hepatocyte necrosis through steatosis in GKO mice treated with high-dose APAP compared to wild-type controls. A unique feature of the hepatic necrosis in GKO mice was the appearance of microvesicular steatosis as a result of centrilobular necrosis, rather than the inflammation seen in the wild type. The GSH surrogate and general antioxidant ψ-GSH alleviated APAP toxicity irrespective of the Glo-1 status, suggesting that oxidative stress is the primary driver of APAP toxicity. Overall, the exacerbation of APAP hepatotoxicity in GKO mice suggests the importance of this enzyme system in antioxidant defense against the initial stages of APAP overdose.

## 1. Introduction

The local redox state influences inflammatory, metabolic, and proliferative processes in liver disorders [1]. Insufficient antioxidant capacity relative to the magnitude of oxidative insults leads to undesirable damage to major biomolecules. Redox homeostasis is maintained by enzymes (e.g., superoxide dismutase (SOD), catalase, and glutathione-dependent enzyme pathways) and endogenous molecules (e.g., glutathione (GSH), vitamin E, and ascorbic acid). These entities neutralize oxidative insults, such as reactive oxygen species (ROS) produced by metabolic processes and the mitochondrial electron transport chain. Increased oxidative stress leads to an excess of reactive dicarbonyl metabolites, such as methylglyoxal (MEG), a byproduct of glycolysis, and, in turn, results in further ROS generation. Indeed, hyperglycemia is known to increase MEG levels, therefore harming liver function [2]. At higher concentrations, MEG exerts cytotoxicity by covalently modifying proteins, nucleotides, and phospholipids. Alterations in protein structure and function are caused by the MEG-dependent formation of advanced glycation end products (AGEs) and are considered a causative factor behind the resultant dicarbonyl stress [3,4]. Important AGEs derived from MEG are the non-fluorescent products 5-hydro-5-methylimidazolone (MG-H1) and tetrahydropyrimidine (THP), as well as the fluorescent argpyrimidine. AGEs bind to and activate the receptor for advanced glycation end products (RAGE). RAGE is expressed by hepatocytes and hepatic stellate cells. Interactions with ligands cause RAGE to activate various fibrotic, angiogenic, and apoptotic pathways. The expression of inflammatory mediators, such as TNF-α and IL-6, activates hepatic stellate cells [5,6,7].

The glyoxalase system, a major regulator of oxidative stress resulting from dicarbonyl MEG, consists of two crucial enzymes, glyoxalase 1 (Glo-1) and glyoxalase 2 (Glo-2). Glo-1 is a dimeric metalloprotein containing a Zn^+2^ ion in each of the two subunits [4]. Human Glo-1 expression is controlled by activator protein-2α (AP-2α), nuclear transcription factor-κB (NF-κB), early gene 2 factor isoform 4 (E2F4), antioxidant-response element (ARE), and nuclear factor erythroid-2-related factor 2 (Nrf2), among other entities [4]. Glo-1 is an aldoketomutase—it rearranges the hemithioacetal of GSH with MEG into S-D-lactoylglutathione. The latter is hydrolyzed by Glo-2 into relatively non-toxic D-lactate, with the liberation of GSH (Supporting Information, Figure S1) [8]. Decreased levels of Glo-1 and increased intracellular dicarbonyl stress occur in liver fibrosis, cirrhosis, non-alcoholic steatohepatitis (NASH), and hepatocellular carcinoma. The pharmacological modulation of Glo-1 and the silencing of RAGE have shown promise in preclinical models of CCl_4_-induced liver fibrosis and hepatocellular carcinoma, respectively [9]. The Glo-1-mediated detoxification of MEG and structurally related dicarbonyls is considered a rate-limiting step [3]. Therefore, the modulation of the Glo-1/AGE/RAGE axis for the management of chronic liver diseases has been explored by us and many others in the field.

Although the role of Glo-1 and AGE/RAGE in chronic liver diseases is known [3,9], their role in acute liver failure is relatively understudied. Drug-induced liver injury constitutes ~60% of acute liver failure cases. Among these, acetaminophen (N-acetyl-p-aminophenol, APAP)-induced liver toxicity is the leading cause of liver failure in the United States [10]. An estimated 50,000 people are hospitalized annually due to APAP overdose, and up to 500 people die each year. APAP overdose results in the accumulation of high levels of the extremely reactive N-acetyl-p-benzoquinoneimine (NAPQI), which rapidly depletes cellular GSH. This leads to oxidative stress and the accumulation of AGEs, ultimately causing cell damage and death. N-acetylcysteine (NAC) is a classical clinical antidote for APAP overdose. NAC triggers GSH synthesis, enhances glutathione-S-transferase (GST) activity, and facilitates detoxification by scavenging ROS. Reduced GSH levels are observed after APAP overdose in models of APAP toxicity and also in patient serum [11,12]. Being a coenzyme for Glo-1, the deficiency of GSH is expected to perturb the Glo1/AGE/RAGE axis. It is, therefore, essential to study the effects of GSH depletion and Glo-1 inactivation on APAP-induced liver toxicity. The pharmacological modulation of Glo-1 and gene deletion are expected to shed light on the complex interplay between these pathways involved in hepatotoxicity and provide insights into potential therapeutic interventions for APAP overdose.

Toward that goal, we used constitutive Glo-1-knockout mice to decipher the role of Glo-1 in APAP-induced hepatotoxicity. Previously, we described a bioavailable GSH surrogate, ψ-GSH, as a potential antidote for APAP liver toxicity [13]. The utility of GSH itself or GSH precursors as antidotes is hampered by the cleavage of the γ-glutamylcysteinyl (Glu-Cys) amide bond in GSH by the ubiquitous γ-glutamyltranspeptidase. Designed to resist this enzyme, a synthetic GSH analogue, ψ-GSH, was developed by us by substituting the Glu−Cys amide bond with a ureide isostere. ψ-GSH is able to substitute for GSH in GSH-dependent enzymatic systems, including Glo-1. It is also transported in the cells by a GSH transport mechanism. ψ-GSH mitigates APAP toxicity and improves survival, even when administered post-APAP overdose, mimicking the clinical scenario in a mouse model of APAP hepatotoxicity. It also replaces GSH in major GSH-dependent enzymatic pathways [14]. Thus, the innate antioxidant potential of ψ-GSH and its ability to supplement antioxidant enzymatic functions could be responsible for its hepatoprotective effect. We have used ψ-GSH to study the central function of Glo-1 in neurodegenerative pathology and evaluated its potential as a therapeutic for the management of neurodegenerative disorders, such as Alzheimer’s disease [14,15]. Here, we used ψ-GSH as a pharmacological tool for the characterization of the Glo-1 pathway in APAP-induced liver toxicity.

In this study, we created an APAP hepatotoxicity model in whole-body Glo-1-knockout (Glo-1^−/−^, GKO) mice and examined the effects of ψ-GSH on this model [16]. Increased liver AGEs and reduced anxiety-like behavior were previously noted in GKO mice compared to age-matched wild-type mice. We observed the exacerbation of liver toxicity induced by high-dose APAP in GKO mice. Histological analysis suggested an unexpected and distinct pattern of hepatocyte necrosis in GKO mice when compared to wild-type (WT) mice, *through steatosis rather than inflammation*. APAP metabolism was also significantly altered in the knockout mice. Biochemical analysis confirmed the involvement of the Glo-1/AGE/RAGE axis in GKO mice. Intervention with ψ-GSH mitigated oxidative stress in both WT and GKO mice. This study, for the first time, describes the significance of the Glo-1 pathway in APAP-overdose-induced oxidative stress.

## 2. Materials and Methods

### 2.1. Materials

Acetaminophen (Spectrum Chemical Corp, New Brunswick, NJ, #AC100, USA), triethanolamine (Sigma-Aldrich Corp, # t58300, St Louis, MI, USA), 2-vinylpyridine (Alfa Aesar, Ward Hill, MA, #A14056, USA), RIPA buffer (Cayman Chemical Co., Ann Arbor, MI, # 10010263, USA), GSH MES buffer (Cayman Chemical Co., # 703010, Ann Arbor, MI, USA), and cOmplete Protease Inhibitor Cocktail (Roche, Penzberg, Germany, #4693124001) were used in this study.

### 2.2. Animals

C57BL/6 male and female mice aged 18–20 weeks were obtained from Charles River Laboratories and used in the study. Sperm carrying the Glo1^tm1a(KOMP)Mbp^ allele was obtained from the European Mouse Mutant Achieve (EM:09893), and live mice were produced via in vitro fertilization at the University of Minnesota Mouse Genetics Laboratory. The resulting heterozygous mice were mated to generate homozygous GloKO mice (GKO) and maintained in the Lee lab at the University of Minnesota. All experimental procedures and animal handling were conducted in accordance with the national ethics guidelines and complied with the Institutional Animal Care and Use Committee (IACUC) protocol requirements of the University of Minnesota (Minneapolis, MN, USA). Every effort was made to minimize animal suffering and the number of animals used in this study. The animals were housed in a university facility in groups of four per cage under controlled environmental conditions in a 12 h/12 h light/dark cycle and were allowed access to food and water ad libitum. The experimental procedures were carried out during the light phase of the light/dark cycle.

### 2.3. Behavioral Assessment of GKO Mice

Twelve-week-old WT and GKO male and female mice were used for behavior analysis. For the open-field test (40 cm × 40 cm × 40 cm), the mice were habituated for an hour in a dark recording room. After that, each mouse was put in the center of an open field and video-recorded for 10 min, and the first 5 min were analyzed. For the light–dark box test, after habituation for an hour in the test room, each mouse was placed in the center of the lighted zone, facing toward the maze wall, and was recorded for 15 min, and the first 10 min were analyzed. For the tail-flick test, the mice were habituated for an hour in the recording room. Mice tails were exposed to a light beam centered on a ventral surface, about 15 mm from the tip of the tails, and the latency for withdrawal of the tail was recorded for each mouse. In the T-maze test, a spontaneous alternation protocol was used for the assessment of working cognitive behavior. After habituation for an hour in the testing room, the mice were allowed to freely explore the entire maze. This was followed by confinement in the start arm for 30 s and 15 free-choice trials to calculate percentage alternations.

### 2.4. Animal Handling

The mice were allowed to acclimatize to the facility for at least 1 week prior to the start of the experiment. Prior to drug administration, C57BL/6 and GKO mice were fasted overnight, weighed, and randomly assigned to different treatment groups (*N* = 10–12 per experiment). Acetaminophen was dissolved in warm saline and injected intraperitoneally (i.p.) at a dose of 250 mg/kg (1.65 mmol/kg). At the designated time, either ψ-GSH (800 mg/kg) or saline was administered intraperitoneally to the mice, with control groups receiving only saline. Food was returned to the cages after the final drug injection, and the animals were monitored every 30 min for the first 2 h. At the end of the experiment, blood and liver tissue samples were collected from the mice at 24 h after APAP administration. For APAP metabolite quantification at the early stages of toxicity, overnight-fasted C57BL/6 male and female mice were injected with APAP (250 mg/kg, i.p.), and blood samples were withdrawn at 0, 0.5, 1, 2, 4, and 6 h (*N* = 4 per time point). Additionally, liver tissues from C57BL/6 and GKO male mice (*N* = 4) were collected 1 h after APAP overdose to measure the liver concentrations of APAP metabolites. Liver tissues were immediately frozen and stored at −80 °C until further use or fixed in 10% neutral buffered formalin for histological analysis.

### 2.5. ALT Level Assessment in Serum

Serum alanine aminotransferase (ALT), creatinine, and creatinine kinase (CK) measurement was used to assess liver and kidney damage caused by APAP overdose. Additionally, the levels of cholesterol and triglycerides were measured. Blood samples were collected from the mice at 24 h after APAP overdose. The clotted blood was centrifuged to obtain serum, which was then stored on ice until it was submitted to the Veterinary Diagnostic Laboratory at the University of Minnesota for blood chemistry measurement.

### 2.6. Histology

Liver morphology was evaluated by the histological analysis of H&E-stained liver sections. The liver harvested from the mice at 24 h after APAP overdose was immediately fixed with 10% formalin. The fixed liver samples were then submitted to the Comparative Pathology Shared Resource at the University of Minnesota for processing. For the immunohistochemical analysis of adipose-differentiation-related protein (ADFP), 4 μm of formalin-fixed, paraffin-embedded tissue sections were deparaffinized and rehydrated, followed by antigen retrieval using a low-pH citrate buffer. After quenching endogenous peroxidase, immunohistochemistry was performed using a rabbit polyclonal primary antibody (Thermo Scientific, PA1-16972, Waltham, MA, USA) diluted to 1:100 and incubated for 30 min at room temperature. Antibody binding was detected using the Rabbit Envision (Dako) secondary antibody. Diaminobenzidine was used as the chromogen, and Mayer’s hematoxylin (Dako) was used as the counterstain. Primary antibodies were substituted with appropriate negative control IgG for negative control slides.

### 2.7. Measurement of Lipid Content

The total liver lipid content was determined using sulfo-phospho-vanillin assay, as described previously, with slight modifications [17]. The liver homogenates (100 μL of 4 mg/mL protein content) were extracted twice with 500 µL of 2:1 *v*/*v* chloroform/methanol. The combined organic phase containing lipids was dried in vacuo and then redissolved in chloroform. A fraction of the chloroform fraction was dried before incubation with 100 µL of concentrated H_2_SO_4_ for 20 min at 90 °C. The samples were then immediately cooled on ice and transferred to a 96-well plate. Next, 60 µL of 0.2 mg/mL vanillin reagent prepared in 17% phosphoric acid was added to these samples, and the absorbance at 540 nm was measured. The total lipid content was calculated using a standard curve of oleic acid prepared under similar conditions.

### 2.8. APAP and Metabolites in Serum and Liver Samples

Mouse serum samples collected, as described in the ALT assessment, were used for the measurement of APAP and metabolites by mass spectrometry. Serum and liver homogenates were processed by the addition of 90 μL of acetonitrile to 10 μL of the serum sample. The samples were mixed by vortexing for 30 s and then centrifuged at 21,130× *g* for 5 min at 4 °C. The supernatants were collected and transferred to a fresh 1.5 mL microcentrifuge tube.

Liquid chromatography–tandem mass spectrometry (LC-MS/MS) analysis was conducted using an integrated system consisting of an Agilent 1260 High-Performance Liquid Chromatography (HPLC) device (Agilent Technologies, Santa Clara, CA, USA) coupled with an AB Sciex QTRAP 5500 mass spectrometer (AB Sciex LLC, Toronto, ON, Canada), with slight modifications to the method described previously [18].

Briefly, chromatographic separation of the samples was achieved using a Thermo Aquasil C18 column (150 × 2.1 mm, 3 μm). A binary mobile phase system at a constant flow rate of 0.3 mL/min was used with mobile phase A consisting of 0.1% formic acid in water and mobile phase B consisting of 0.1% formic acid in acetonitrile. The gradient elution profile began with a linear increase from 0% to 5% B over 2 min. Subsequently, the gradient transitioned from 5% to 80% B over another 2 min, held at 80% B for 1.8 min, and then returned to 0% B over 0.2 min. Finally, the column was equilibrated at 0% B for 6 min. Only desired fractions of eluates, corresponding to the 1.3 to 10 min retention time window, were collected for subsequent LC-MS/MS analysis. Samples were introduced into the mass spectrometer via electrospray ionization in a fast polarity-switching mode. The instrument parameters were set as follows: curtain gas at 25 psi, collision-activated dissociation (CAD) gas at medium flow, ion spray voltage at 5000 V or −4500 V, source temperature at 650 °C, gas 1 at 60 psi, and gas 2 at 50 psi. For targeted quantitation, MRM was used by monitoring specific mass transitions for each analyte, as confirmed by the commercial standard of each metabolite, and the transitions are listed as follows: *m*/*z* 152.1 → 110.1 (APAP), *m*/*z* 182.1 → 108.0 (APAP−OMe), *m*/*z* 271.1 → 140.0 (APAP−Cys), *m*/*z* 313.1 → 208.0 (APAP−NAC), *m*/*z* 457.1 → 328.1 (APAP−GSH) in positive mode and *m*/*z* 230.0 → 150.0 (APAP−Sulf), and *m*/*z* 326.0 → 150.0 (APAP−Gluc) in negative mode.

### 2.9. Measurement of Liver GSH Content

The GSH/GSSG ratio was determined by following the protocol included in the glutathione assay kit (Cayman Chemical Co., # 703002, Ann Arbor, MI, USA). Briefly, liver tissues were harvested, rinsed with PBS, and homogenized using 2 mL of 50 mM MES buffer containing 1 mM EDTA (pH 6.0) per gram. The supernatant was collected after centrifugation at 10,000× *g* for 15 min at 4 °C. Deproteinization was carried out by adding an equal amount of 10% metaphosphoric acid. The deproteinized homogenate (100 μL) was neutralized with 5 μL of 4 M triethanolamine solution and diluted 150 times with MES buffer. To measure the reduced GSH (GSSG), deproteinated samples were treated with 1 M 2-vinylpyridine solution (10 μL) and allowed to incubate for an hour at room temperature. GSH and GSSG levels were determined from the absorbance after reaction with 5-thio-2-nitrobenzoic acid at 412 nm as per the manufacturer’s instructions.

### 2.10. Lipid Peroxidation Assay

Hepatic lipid peroxidation was measured by TBARS assay. The levels of malondialdehyde (MDA), a breakdown product of lipids, were determined colorimetrically using the TBARS assay kit (Cayman Chemical Co., Cat. No. 10009055, Ann Arbor, MI, USA), as described previously.

### 2.11. Evaluation of Protein Carbonyl Content

The level of the protein carbonyl present in the liver samples was measured using the protein carbonyl colorimetric assay kit (Cayman Chemical Co., Cat. No. 10005020, Ann Arbor, MI, USA) and the Dot Blot assay kit (Abcam, Cat. No. ab178020, Waltham, MA, USA). For dot blot analysis, liver homogenates were first derivatized with DNPH as per the manufacturer’s instructions. A total of 50 ng of the derivatized protein was used to determine the level of the protein carbonyl in each group.

### 2.12. Determination of CYP2E1 Activity

CYP2E1 activity was measured using *p*-nitrophenol as a substrate and by following its oxidation to *p*-nitrocatechol in the presence of NADPH [19]. Liver homogenates (200 μg) from WT and GKO mice were mixed with 100 mM potassium-phosphate buffer (pH 7.4) containing 0.2 mM PNP (in 100 µL of reaction). The reaction was started by the addition of 1 mM NADPH and incubation for 60 min at 37 °C. After quenching the reaction with 20% trichloroacetic acid, the supernatant was neutralized by the addition of 10 M sodium hydroxide. The absorbance at 546 nm was used to determine CYP2E1 activity using the formula OD_546_/9.53/0.2/60/7.1 × 10^6^ (pmol/min/mg of protein).

### 2.13. Determination of Liver Superoxide Dismutase (SOD) Activity

The SOD activity of liver homogenates (containing 50 mM MES, pH 6.5, 1 mM EDTA) was determined using the commercial SOD assay kit (Cayman Chemical Co., # 66970752, Ann Arbor, MI, USA). Briefly, 10 µL of the liver homogenate was treated with 200 µL of the radical detector solution provided in the kit. The reaction was initiated by adding 20 µL of xanthine oxidase, and the rate of change in the absorbance at 450 nm was used to calculate the SOD activity per sample.

### 2.14. Determination of Liver Catalase Activity

To determine catalase activity, the procedure described in the catalase assay kit (Cayman Chemical Co., # 66970752, Ann Arbor, MI, USA) was followed. Briefly, the liver homogenate was treated with 30 µL of methanol, and the reaction was initiated by the addition of H_2_O_2_. The formaldehyde produced was measured by the reaction with 4-amino-3-hydrazino-5-mercapto-1,2,4-triazole (Purpald). The complex upon oxidation with potassium periodate produces a purple color, which was measured by the absorbance at 540 nm to calculate the catalase activity of the sample, as described in the kit.

### 2.15. Determination of AGE Levels by AGE ELISA

Total AGEs in the liver tissues were measured using a commercial AGE ELISA kit (XpressBio, Cat. No. XPEM0716, Frederick, MD, USA). Briefly, 100 µL of the liver homogenate (diluted 400-fold with dilution buffer) was added to a 96-well plate pre-coated with a capture anti-AGE antibody and allowed to incubate at 37 °C for 90 min. Following incubation and washing, 100 µL of a biotin-labeled anti-AGE antibody was added to each well. After another round of washing, HRP-conjugated streptavidin (100 µL) was added, and incubated for 30 min at 37 °C. The plate was washed again and incubated with TMB substrate for 20 min before quenching the reaction with the provided solution, and absorbance was measured at 450 nm, reflective of the AGE content.

### 2.16. Western Blot Analysis

Parts of livers were lysed with RIPA buffer containing protease inhibitors (Roche, New York, NY, USA). Protein concentrations were determined using the BCA Protein Assay Kit (Thermo Fisher Scientific, Waltham, MA, USA). The proteins were denatured, and equal amounts of proteins (a total of 40 µg/lane) from different groups, as indicated in the figures, were separated by SDS-PAGE and transferred to a polyvinylidene fluoride (PVDF) membrane via the wet transfer method for immunoblot analyses. The PVDF membrane was blocked for 2 h using 5% fat milk and incubated with the antibody to ADFP (#PA1-16872), cleaved caspase-3 (#9661), RIPK3 (#95702), p62 (#5114), RAGE (ab3611), AGE (ab9890), Glo-1 (ab137098), Glo-2 (AF5944), HMGB1 (3935S), MG-H1 (STA-011), Bax (sc-20067), and the antibody to α-tubulin (ab4074) at 4 °C overnight. Primary antibodies were labeled with horseradish-peroxidase-conjugated secondary antibody (1:5000) and ECL substrates (Bio-Rad, Mississauga, ON, Canada). The densitometric intensities of the bands were quantified using ImageJ software (version 1.54g).

### 2.17. Statistical Analysis

Statistical analysis was performed using GraphPad Prism ver. 10. Data were expressed as the mean ± standard error of the mean. All data were analyzed using one- or two-way analysis of variance (ANOVA), with Tukey’s or Sidak’s post hoc test or Student’s *t*-test, wherever appropriate. Statistical significance was held at *p* < 0.05.

## 3. Results and Discussion

### 3.1. Liver Toxicity Induced by APAP Overdose Is Potentiated in Glo-1-Knockout Mice

A single high dose of APAP has been used to generate a mouse model that enabled the study of toxicological mechanisms, as well as protective interventions. We have previously studied the effect of potential antidotes for APAP overdose in fasted Swiss Webster mice [13,18]. This study evaluated the role of the Glo-1 pathway in APAP-induced hepatotoxicity, for which we used constitutive knockout of Glo-1 (Glo-1^−/−^, GKO) in a C57BL/6N background. Due to the higher susceptibility of C57BL/6N mice over C57BL/6J mice to APAP toxicity [20], GKO mice were maintained in a 6N background. The behavioral characterization of GKO mice has been reported previously [16]. We confirmed the protein expression of Glo-1 and Glo-2 in GKO mice by Western blot (Supporting Information, Figure S2) and behavioral phenotype in open-field, light–dark box, T-maze, and tail-flick tests. A significant behavioral impact of Glo-1 was noted only in GKO male mice in the open-field and light–dark box tests, which is in agreement with previous reports claiming a correlation between the Glo-1/MEG concentration and anxiety-like behavior [21,22]. GKO mice spent more time in the center zone of the open-field box (Supporting Information, Figure S3A) and in the lit area of the light–dark box (Supporting Information, Figure S3D,E), indicating anxiolytic behavior. The mice did not show signs of motor deficits based on the total distance moved (Supporting Information, Figure S3B). No change in exploratory behavior was seen in the T-maze spontaneous alternation test (Supporting Information, Figure S3C). Additionally, the response to heat stimuli in the tail-flick test did not differ between WT and GKO mice (Supporting Information, Figure S3F).

**Figure 1:**
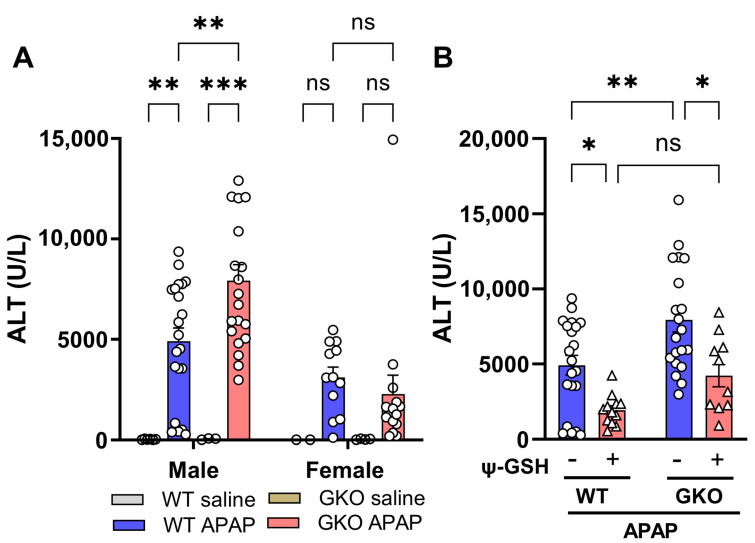
The effect of Glo-1 deletion on hepatotoxicity induced by high-dose APAP. (**A**) Serum samples were collected 24 h after intraperitoneal administration of APAP (250 mg/kg) from C57BL/6 male and female mice to determine ALT levels. Significant elevation of serum ALT levels was noticed in GKO male mice compared to WT mice, while female mice were resistant to this APAP-induced ALT elevation. (**B**) Serum ALT levels in male mice pretreated with ψ-GSH (800 mg/kg, i.p.) 30 min prior to injection of APAP (250 mg/kg, i.p.). Significant protective effect of ψ-GSH treatment was observed in WT-APAP and GKO-APAP groups. The results presented are a combination of two different experiments (* *p* < 0.05; ** *p* < 0.01; *** *p* < 0.001; ns not significant; two-way ANOVA followed by Tukey’s post hoc multiple-comparison test).

We first calibrated a dose of APAP that causes reproducible and measurable liver enzyme elevation, such as serum alanine aminotransferase (ALT), in GKO mice as a gauge of liver toxicity. The dose equivalent to our previous studies (370 mg/kg) created a much higher response, with an effect on animal viability. Thus, we selected a 250 mg/kg dose in this study, which produced significant ALT elevation without affecting survival. This dose caused more than 120-fold elevation in the ALT levels of the APAP-treated mice when compared to the ALT levels in the saline-treated animals (WT-APAP, 4906.52 ± 662.16 vs. WT-saline, 40.00 ± 6.86 U/L; Figure 1A). A dramatic increase in ALT levels was observed in APAP-treated GKO mice (7919.80 ± 799.07 U/L) over the matched saline-treated controls (56.33 ± 16.48 U/L) and, most noticeably, over WT-APAP mice (1.6-fold). Additionally, the change in the ALT level was significant only in male mice compared to female mice. Treatment with ψ-GSH showed significant hepatoprotection by alleviating the increased ALT levels induced by APAP overdose in WT mice, as previously reported by us in an independent study [13], and in GKO animals (Figure 1B). Designed as a metabolically stable analog of GSH, ψ-GSH, with its inherent antioxidant nature due to aliphatic thiol, is capable of non-enzymatic neutralization of ROS generated by APAP, which could explain the hepatoprotection offered by ψ-GSH in APAP-treated GKO and WT mice.

**Figure 2:**
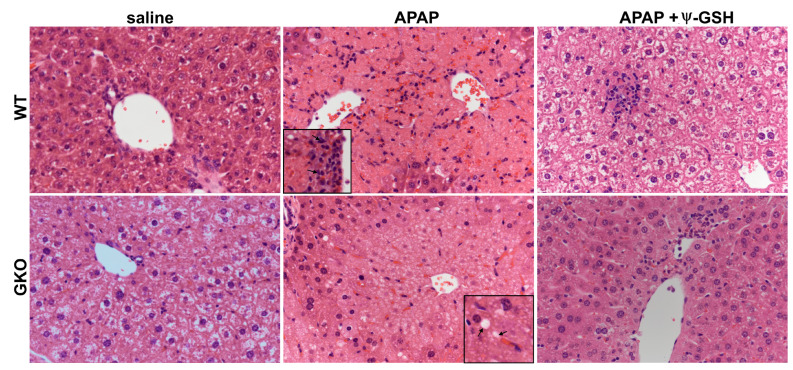
Histological examination of the liver tissue from WT and GKO male mice exposed to APAP overdose. Livers of saline-treated WT and GKO mice showed intracytoplasmic vacuoles (clear cytoplasm, physiological). APAP treatment resulted in coagulative necrosis in the centrilobular zone, with a similar extent of necrosis in both WT and GKO mice. Inflammatory cells were observed surrounding the central vein in WT-APAP mice (inset, top row), while necrotic hepatocytes showed prominent microvesicular lipid accumulation in the GKO-APAP group as indicated by black arrows (inset, bottom row). Ψ-GSH treatment reversed histological changes caused by APAP overdose.

Histopathological evaluation of liver tissues (Figure 2) corroborated the gender-specific findings related to ALT elevation. Female mice treated with high-dose APAP showed low-to-moderate necrosis (score 1–2) of the hepatocytes irrespective of the Glo-1 status. In male mice, the WT-APAP group showed coagulative necrosis (score 3–4) covering 30–60% of the centrilobular-to-midzonal area, with markedly swollen hepatocytes (inflammation score 2–3), as reported by others [23,24]. APAP-treated GKO mice, in addition to necrosis (score 3–4) and swollen hepatocytes (inflammation score 1), exclusively showed intracytoplasmic, empty-to-pale-pink discrete vacuoles, indicative of microvesicular lipid accumulation (Figure 2, inset, bottom row). Such microvesicular steatosis, a type of hepatocellular degeneration, was severe in the centrilobular zone of APAP-treated GKO mice (score 3–4). Previously, Spanos et al. [25] also reported a dramatically increased level of intracellular lipids and MEG, accompanied by decreased Glo-1 protein expression, in HepG2 cells treated with oleic acid [25]. Additionally, the reduced reservoir of GSH due to a lack of Glo-1 expression makes steatotic hepatocytes more susceptible to APAP-induced hepatotoxicity [26]. Treatment with ψ-GSH effectively reduced the extent of necrosis and inflammation in both WT and GKO mice.

**Figure 3:**
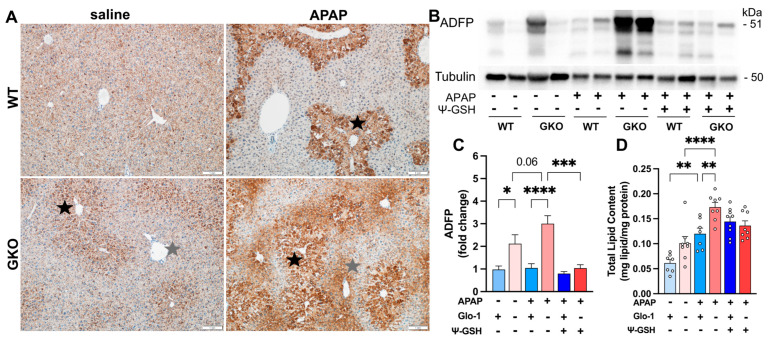
Deletion of Glo-1 exacerbates microvesicular steatosis induced by high-dose APAP. (**A**) Representative images of anti-ADFP immunostaining in the liver tissues of saline- and APAP-treated WT and GKO male mice. Mild centrilobular staining (black star) seen in the GKO-saline animals progressed to diffuse, marked centrilobular ADFP staining in the GKO-APAP group, with moderate periportal (gray star) staining. (**B**) Western blot analysis of the ADFP expression in liver homogenates showed the highest levels in the GKO-APAP group relative to GKO-saline and WT groups. (**C**) Quantitation of the blots shown in (**B**) using α-tubulin as a housekeeping protein control. (**D**) Total lipid quantitation in liver homogenates using sulfo-phospho-vanillin assay (* *p* < 0.05; ** *p* < 0.01; *** *p* < 0.001; **** *p* < 0.0001; one-way ANOVA followed by Sidak’s post hoc multiple-comparison test).

The presence of microvesicular steatosis was further confirmed by examining the levels of adipose-differentiation-related protein (ADFP) in the liver using immunohistochemical and Western blot analyses. ADFP (also known as perilipin-2) is localized to the surface of lipid droplets that comprise primarily a neutral lipid core with an outer phospholipid monolayer [27]. The abundance of ADFP is directly associated with lipid accumulation. Immunohistochemical analysis (Figure 3A) showed higher anti-ADFP immunoreactivity in APAP-treated mice compared to their saline-treated counterparts. A markedly higher level of ADFP was seen in the GKO-APAP group compared to the WT-APAP group. Diffuse, panlobular mild ADFP staining was apparent in WT-saline mice, while clear zonal distribution of anti-ADFP immunoreactivity was observed in WT-APAP, GKO-saline, and GKO-APAP groups. Specifically, ADFP staining was most intense in the centrilobular regions (black star) as compared to the periportal regions, which is consistent with the increased light microscopic pathology observed in the H&E-stained sections (Figure 2). Immunoblot analysis of liver homogenates further confirmed these observations (Figure 3B,C). ADFP protein expression was higher in both saline- and APAP-treated GKO mice compared to the corresponding WT groups. APAP treatment significantly increased ADFP levels in GKO mice, suggesting exacerbation of APAP-associated steatosis by Glo-1 deletion. ψ-GSH treatment significantly attenuated the increased ADFP expression in GKO mice.

To investigate lipid changes in GKO mice treated with APAP, we examined serum cholesterol and triglyceride levels. No significant differences were noted in WT and GKO mice in the presence and absence of APAP (Supporting Information, Figure S4). However, a noticeable increase in creatinine (~5.7-fold) and creatinine kinase (~2.3-fold) was observed in GKO-APAP mice compared to APAP-treated WT mice. This observation is in line with the prevalence of chronic kidney disease in patients with hepatic steatosis, which shows the association of serum creatinine with hepatic steatosis risk [28]. Biochemical analysis of the total lipid content in liver homogenates using sulfo-phospho-vanillin assay showed a higher lipid content in the APAP-treated mice compared to the respective saline controls, with the highest levels found in the GKO-APAP group (Figure 3D). The total liver lipid content remained unaffected by ψ-GSH treatment. Exacerbation of intracellular lipid accumulation is proposed to contribute independently to non-alcoholic fatty liver disease and chronic kidney disease [29]. Collectively, these observations indicate the exacerbation of APAP hepatotoxicity in GKO mice compared to WT animals in a degenerative manner by a unique mechanism involving hepatocellular steatosis.

### 3.2. Deletion of Glo-1 Reduced Overall Metabolic Detoxification of APAP

**Figure 4:**
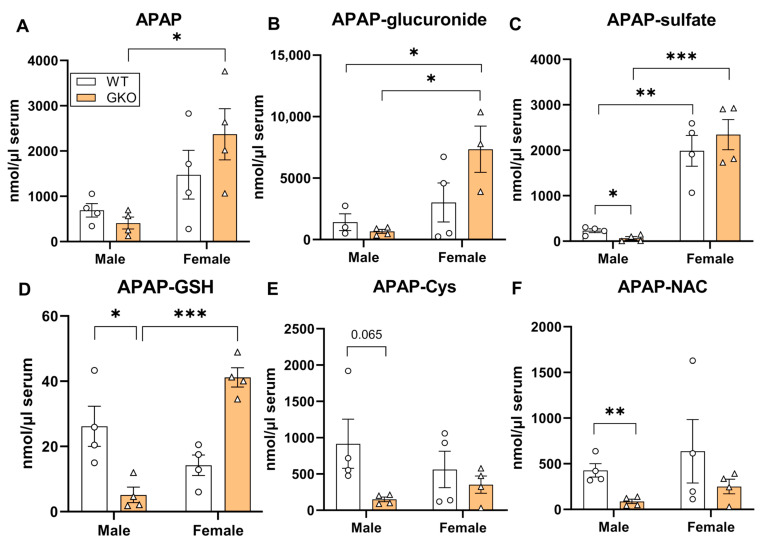
Effect of Glo-1 knockout on the levels of APAP and its metabolites in serum. Serum samples from fasted C57BL/6 male mice collected 24 h after administration of APAP overdose were subjected to metabolite identification by LC-MS/MS. Concentrations of each APAP metabolite from WT and GKO mice are displayed as the mean ± SEM. (**A**) APAP, (**B**) APAP-glucuronide, (**C**) APAP-sulfate, (**D**) APAP glutathione adduct (APAP-GSH), (**E**) APAP cysteine adduct (APAP-Cys), and (**F**) APAP N-acetylcysteine adduct (APAP-NAC) (* *p* < 0.05; ** *p* < 0.01; *** *p* < 0.001; two-way ANOVA followed by Tukey’s post hoc test).

At the therapeutic dose, the majority of the administered APAP is metabolized by phase II metabolism by conjugation to glucuronide and sulfate metabolites [30]. A small portion (5–10%) is oxidized by microsomal CYP2E1 and CYP1A2 to a highly reactive intermediate, N-acetyl-p-benzoquinone imine (NAPQI) [31]. Hepatic GSH effectively quenches the toxic NAPQI under normal homeostasis. However, under disease conditions or accidental overdose, wherein the supply of GSH is limited, NAPQI exerts toxic effects by irreversibly modifying cysteine residues in cellular proteins, exacerbating the oxidative stress and resulting cellular damage [13,32,33,34,35]. Deletion of Glo-1 did not affect the function of CYP2E1 (Supporting Information Figure S6B). To understand the effect of Glo-1 on APAP toxicity, we examined the effect of Glo-1 deletion on the levels of the major metabolites of APAP. Concentrations of individual APAP metabolites in serum samples were determined using LC-MS/MS (Figure 4 and Supporting Information, Table S1). Serum APAP levels were similar in WT and GKO male mice (Figure 4A). A trend toward higher intact APAP levels was noted in WT female mice, while the levels were significantly higher in GKO female mice compared to GKO male mice. APAP-glucuronide concentrations were similar in WT and GKO male mice but were elevated in the respective female mice, with the highest levels found in the GKO-APAP group (Figure 4B). The levels of APAP-sulfate were significantly (Figure 4C) reduced in GKO mice compared to WT mice. However, the effect was significant in male mice, while the female mice showed higher APAP-sulfate levels, partly explaining the resistance of female mice to APAP toxicity. Possibly due to higher oxidative stress and reduced GSH levels, the conjugation of APAP to GSH or cysteine protein thiols (phase II) was significantly reduced in GKO male mice. Thus, resultant levels of non-toxic APAP metabolites, such as APAP-GSH, APAP-Cys, and APAP-NAC conjugates, were markedly decreased over those in APAP-WT mice (Figure 4D–F). Furthermore, reduced APAP hydroxylation (phase I), followed by phase II APAP methylation was observed in GKO mice, although the absolute levels of APAP-OMe were significantly lower in both WT and GKO mice when compared to other APAP metabolites. We analyzed early time points until 6 h after APAP overdose to examine these sex-based differences in WT mice (Supporting Information, Figure S5). Higher levels of APAP-sulfate and APAP-GSH metabolites were observed in female mice, while APAP-glucuronide levels were increased in male mice. Similarly, lower levels of non-toxic APAP metabolites, such as APAP-Cys and APAP-GSH, were found in GKO livers compared to the corresponding WT mice livers (Supporting Information, Figure S6A), suggesting higher oxidative stress in GKO mice. These results indicate ineffective detoxification of APAP in the absence of functional Glo-1, contributing to toxicity of the parent APAP. Table S1 lists the concentrations of individual APAP metabolites in WT and GKO mice.

### 3.3. Increased Oxidative Stress Observed in APAP-Treated Glo-1 KO Mice

**Figure 5:**
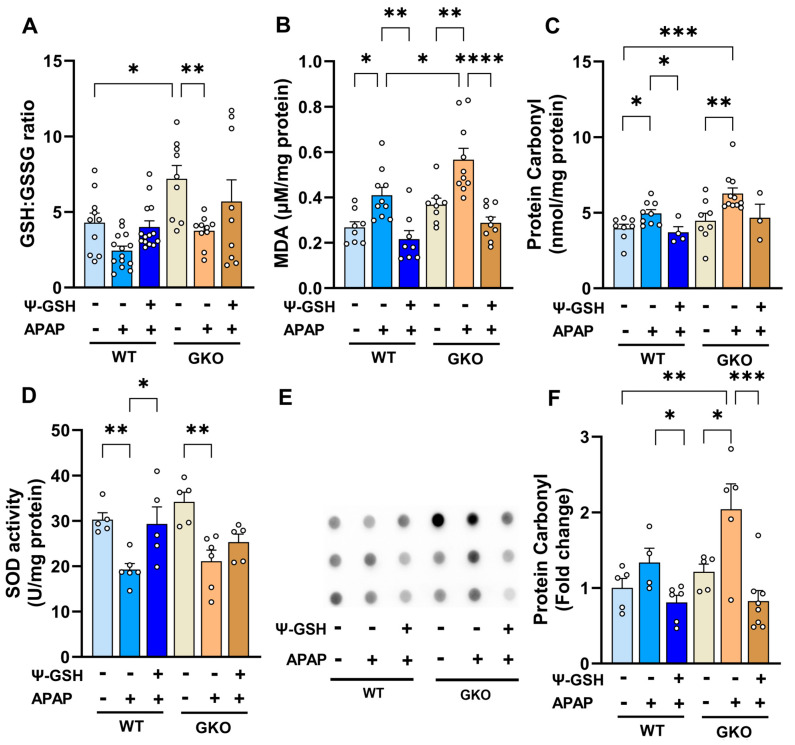
The effect of Glo-1 deletion on the levels of liver oxidative stress markers in APAP-overdosed male mice. All liver tissues for oxidative stress assay were collected at 24 h after overdose of APAP (250 mg/kg, i.p.). ψ-GSH (800 mg/kg, i.p.) was administered 30 min prior to APAP injection. (**A**) Liver GSH/GSSG ratio, (**B**) liver malondialdehyde (MDA) levels (μM per mg protein), (**C**) liver protein carbonyl (nmol per mg protein), (**D**) superoxide dismutase (SOD) activity (U/mg protein), and (**E**) dot blot analysis of protein carbonyls in liver tissue homogenates and its quantitation shown in (**F**) (* *p* < 0.05; ** *p* < 0.01; *** *p* < 0.001; **** *p* < 0.0001; one-way ANOVA followed by Sidak’s post hoc multiple-comparison test).

Oxidative stress has been widely reported as a factor responsible for liver necrosis upon APAP overdose in multiple animal models. Glyoxalase is the major pathway for the detoxification of oxidized sugar metabolites and is intricately involved in the oxidative stress mediated by AGE formation. It is thus expected that the inherent stress produced by the deletion of Glo-1 would be compounded by high-dose APAP. Alterations in the biochemical parameters of oxidative stress, such as GSH levels, protein carbonyl content, lipid peroxidation, and activities of antioxidant enzymes like superoxide dismutase (SOD) and catalase, were used as indices of liver oxidative stress. Due to the clear hepatotoxic phenotype offered by male mice, only male mice were analyzed for changes in biochemical parameters. Similar to our previous reports, we observed attenuation of the redox potential in the livers of APAP-treated WT mice (Figure 5A). Surprisingly, GKO-saline mice showed a significantly higher redox (GSH/GSSG) ratio compared to WT-saline mice (GKO-saline mice, 7.20 ± 0.86 vs. WT-saline mice, 4.29 ± 0.64). This could be attributed to a compensatory mechanism exhibited by GKO mice to overcome the non-functional Glo-1 pathway [36,37]. APAP treatment reduced the GSH/GSSG ratio in WT and GKO mice, which was reversed significantly in the WT group by ψ-GSH (*p* < 0.01, *t*-test). Biochemical analysis of lipid peroxidation (Figure 5B) showed significantly higher levels of malonaldehyde (MDA), one of the final products of fatty acid oxidation, in APAP-treated mice. This increase was higher in APAP-treated GKO mice compared to the corresponding WT mice (GKO-APAP mice, 0.57 ± 0.05 μM/mg protein vs. WT mice, 0.41 ± 0.04 μM/mg protein), which corroborated with the microvesicular steatosis observed in these mice indicative of an imbalance in lipid biology. A similar trend was also observed in the protein carbonyl content (Figure 5C,E) in APAP-treated animals by biochemical assay (DNPH assay), as well as by dot blot analysis. A trend toward increased protein carbonyls in the GKO-APAP group compared to the WT-APAP animals was apparent (*p* < 0.05, *t*-test). Given the compensatory GSH mechanism exhibited by GKO mice, we decided to study the effect of APAP overdose on non-GSH-dependent antioxidant enzymes. Changes in the activity of important oxidoreductase enzymes like SOD and catalase, which play a crucial role in the antioxidant defense against APAP-induced oxidative stress, were examined. Compared to saline-treated mice, APAP-treated WT and GKO mice showed lower SOD activity (~1.6-fold over respective saline groups, Figure 5D), while almost similar catalase activity (Supporting Information, Figure S7) was observed in all treatment groups. ψ-GSH effectively mitigated the emergent oxidative stress in APAP-treated WT mice. This was evident from the elevation in the redox (GSH/GSSG) ratio, reduced lipid and protein oxidation levels, and increased SOD activity. These results reproduced our previous observations in Swiss Webster mice [13], demonstrating the ability of ψ-GSH to ease the oxidative stress induced by high-dose APAP. The protection offered by ψ-GSH in GKO mice using these indices of oxidative stress was almost equal to that in WT mice, suggesting a major contribution of Glo-1-independent antioxidant mechanisms.

### 3.4. Increased Methylglyoxal-Derived AGE Levels in the Livers of Glo-1-Deleted Mice

**Figure 6:**
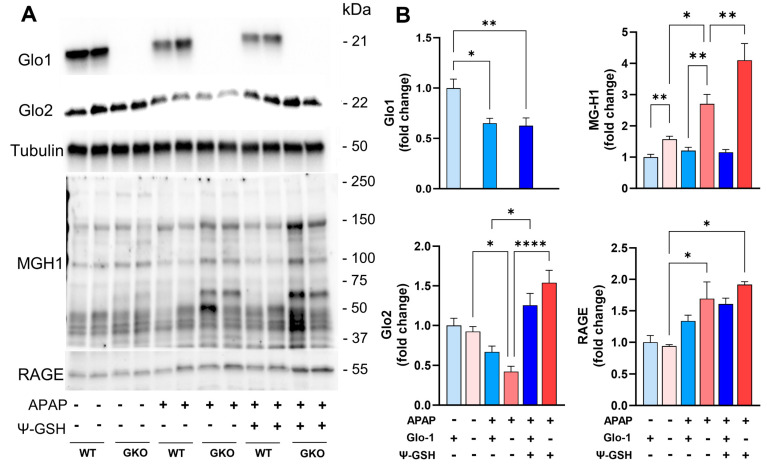
Deletion of Glo-1 increased the levels of MEG and MEG-derived AGEs in the livers of male mice treated with APAP. (**A**) Western blot analysis of Glo-1 and Glo-2 expression, MEG-derived AGE (MG-H1) content, and the expression of RAGE (receptor of AGE). (**B**) Quantification of the blots shown in (**A**). The fold change represents the protein expression relative to α-tubulin, used as a housekeeping protein. Glo-1-related pathway markers AGE and RAGE were significantly elevated in GKO mice livers compared to the corresponding WT mice (* *p* < 0.05; ** *p* < 0.01; **** *p* < 0.0001; one-way ANOVA followed by Tukey’s post hoc test).

We then examined the effect of APAP treatment on Glo-1 expression in WT mice by Western blot analysis (Figure 6). In WT mice, a high dose of APAP reduced the expression of Glo-1 by ~50% (Figure 6A,B). A significant reduction in the Glo-2 level was also observed in APAP-treated WT and GKO mice (Figure 6B). Treatment with ψ-GSH was able to restore Glo-2 expression to the levels in respective saline controls. A compromised Glo pathway in WT mice after APAP overdose could partly explain the equal efficacy of ψ-GSH in WT and GKO mice (Figure 2 and Figure 4).

Glo-1 is involved in the detoxification of MEG using the cofactor GSH. MEG reacts covalently with lysine and arginine residues in proteins, resulting in the formation of irreversible AGEs. Therefore, it is expected that the deletion of Glo-1 would increase MEG levels and, thus, the resultant AGE content in GKO mice. The measurement of AGE levels in the liver tissues of GKO mice has been reported previously by Jang et al. [16]. Western blot analysis using MG-H1 antibody, specific to AGEs derived from MEG, showed significant accumulation of AGEs in APAP-treated GKO livers compared to APAP-treated WT livers (Figure 6A,B). A significant difference in the baseline AGE levels of GKO and WT livers was also noted with MEG-specific MG-H1 antibody (Figure 6B), in agreement with a previous report [16]. We further confirmed Western blot findings using a commercial mouse AGE ELISA kit (Supporting Information, Figure S8). The baseline liver AGE content in saline-treated GKO (14.28 ± 1.87 ng/mg protein) and WT (12.47 ± 3.24 ng/mg protein) mice was similar. The AGE content in GKO-APAP and WT-APAP (42.56 ± 3.53 vs. 34.10 ± 4.32 ng/mg protein, respectively) livers was significantly elevated in comparison with the corresponding saline-treated controls. The measurement of AGE levels using ELISA corroborated Western blot data and showed the highest elevation of AGE levels in GKO-APAP mice, suggesting the contribution of AGE to the exacerbation of APAP toxicity. Treatment with ψ-GSH was ineffective in reducing the AGE content in both WT and GKO mice.

The toxicity induced by AGEs is believed to be through their interaction with the receptor for AGEs (RAGE), which triggers various signaling cascades, leading to further inflammation and oxidative stress. RAGE also plays a significant role in cell death signaling in various pathologies. Thus, we examined the level of RAGE in the livers of APAP-treated animals. Western blot analysis showed significantly increased RAGE levels in APAP-treated groups (1.5- to 1.8-fold), with the highest levels found in APAP-GKO mice (1.8-fold) compared to saline-treated WT controls, as shown in the quantitation in Figure 6B. These data suggest that Glo-1 deletion leads to an elevation in the AGE content upon APAP insult and, thus, resultant cellular toxicity through AGE–RAGE interaction. However, ψ-GSH is unable to correct AGE/RAGE expression after acute treatment, possibly due to the compromised Glo-1 expression after APAP overdose. This further supports our notion that Glo-1 modulates the hepatotoxicity of APAP, as indicated by the increased expressions of individual components of the Glo-1/AGE/RAGE axis.

### 3.5. Distinct Cell Death Mechanisms Activated by High-Dose APAP in the Absence of Glo-1

**Figure 7:**
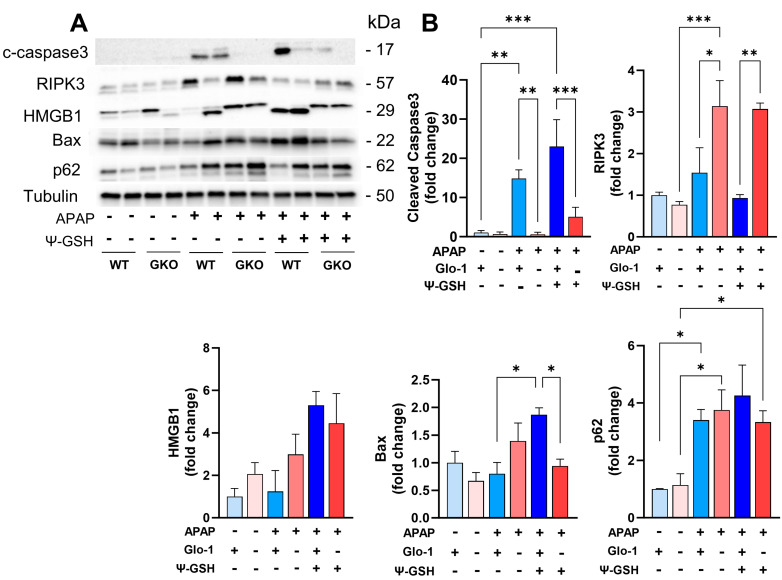
Distinct cell death mechanisms activated by high-dose APAP in the absence of Glo-1 in male mice. The effect of ψ-GSH treatment on these markers is displayed. (**A**) Western blot analysis of cleaved caspase3 (c-caspase3), RIPK3, HMGB-1, Bax, and p62. (**B**) Quantification of blots shown in (**A**). α-Tubulin was used as a housekeeping protein control (* *p* < 0.05; ** *p* < 0.01; *** *p* < 0.001; one-way ANOVA followed by Tukey’s post hoc test).

Given the importance of Glo-1 in the detoxification of MEG, the inhibition of Glo-1 has been used by us and others for the development of anticancer therapeutics [38]. Glo-1 inhibition depresses cell proliferation and induces apoptosis in cancer cells, the degree of which is dependent on the concentration of the generated MEG. Knockout of Glo-1 in human pluripotent cells (hiPSCs) and the liver cell line causes mitochondrial impairment, including diminished membrane potential and dampened respiratory function [39]. In this study, histological analysis (Figure 2) of APAP-treated GKO mouse liver tissue displayed increased microvesicular steatosis, most commonly associated with mitochondrial dysfunction. Additionally, ballooning (swollen) hepatocyte degeneration was apparent in GKO-APAP mice, which is a sign of lipotoxic liver injury. This suggests the activation of distinct cell death mechanisms in GKO mice upon high-dose APAP treatment. We, therefore, investigated the effect of the Glo-1 pathway on APAP-induced cell death. We studied the effect of APAP treatment and Glo-1 deletion on the expression of apoptosis (Bax and caspase-3), autophagy (p62), and necrosis markers (HMGB-1, RIPK3) (Figure 7A). The levels of Bax protein were elevated after APAP treatment, with higher levels found in the APAP-GKO group (2.1-fold over GKO-saline and 1.75-fold over WT-APAP, *p* < 0.05 *t*-test; Figure 7B). In order to determine the consequences of Bax upregulation, we examined the apoptosome marker caspase-3. Although intact procaspase-3 could not be detected in these samples, cleaved caspase-3 was visible in the WT-APAP group, although only upon high exposure, and not in the APAP-GKO group. This observation, coupled with the absence of apoptotic morphology in APAP-treated groups, suggests minimal contribution of apoptosis to APAP-induced cell death irrespective of Glo-1 expression. We also noted that caspase activation can occur in non-apoptotic cell death and may even mediate specific types of necrosis [40]. This observation supports the general knowledge in the field that describes that APAP-induced hepatotoxicity does not involve apoptotic cell death [41] and that caspase inhibitors are ineffective as therapeutic antidotes. Similarly, analysis of the autophagy substrate p62 showed higher accumulation of p62 protein in APAP-treated samples in both WT and GKO mice (3.4- to 3.8-fold compared to the corresponding saline controls), indicating the inhibition of autophagy by high-dose APAP. Experimental NAFLD models and human patients display compromised autophagy and, thus, are suggested to have a higher risk of APAP-induced liver toxicity [42].

The consensus in the field is that APAP liver toxicity stems largely from necrosis or necroptosis [41]. We examined the expression of necrosis and necroptosis markers: high-mobility group box 1 (HMGB1) and receptor interacting protein kinase 3 (RIPK3), respectively. Different isoforms of HMGB1 were observed in APAP-treated livers in the presence and absence of Glo-1, which could indicate different conditions of its release, including different post-translational modifications or proteolytic cleavage. These modifications could contribute differently to the inflammatory response generated by HMGB1. Indeed, the loss of integrity of plasma and the nuclear membrane results in the release of the pro-inflammatory form of HMGB1. Higher inflammation seen in WT-APAP livers indicates early release of HMGB1 in these mice [43], which corresponds to a higher expression of cleaved HMGB1 in these mice compared to that in the WT-saline group. The intensity of HMGB1 bands was higher in the saline-treated GKO livers, which was further elevated upon exposure to high-dose APAP. A change in the HMGB1 isoform released in APAP-treated GKO and WT mice, possibly due to proteolytic cleavage, is expected to affect its translocation and release and could be responsible for differences in the inflammatory response. We further examined the expression levels of RIPK3, which is reported to be a molecular switch from apoptosis to necroptosis in APAP hepatotoxicity [44]. APAP treatment increased RIPK3 protein levels in both WT and GKO mice, while the highest increase was seen in GKO-APAP mice compared to GKO-saline mice (4.1-fold) and the WT-APAP group (2.1-fold). HMGB1 is known as a prototypic-damage-associated molecular pattern (DAMP) involving the engagement of cytokines and cell surface receptors, such as RAGE. It is implicated in the amplification of hepatocyte necrosis via an RIPK3-dependent pathway [45]. It is also interesting to note that accumulated p62 has been shown to interact with RIPK1 and RIPK3, facilitating the creation of necrosomes and, ultimately, necroptosis [46]. Treatment with ψ-GSH did not show any significant effect on the expression of proteins involved in these cell death mechanisms irrespective of the Glo-1 status of the mice.

## 4. Discussion

In this study, we examined the role of Glo-1 in acute-APAP-overdose-induced liver injury using a constitutive Glo-1-deleted mouse model. We also examined the effect of a synthetic Glo-1 cofactor surrogate and a general antioxidant, ψ-GSH, on this model. Our results indicate that mice lacking Glo-1 expression show a distinct pattern of hepatotoxic effects after APAP overdose when compared to WT mice. This finding is corroborated by elevated oxidative stress, APAP metabolism, liver chemistry/histopathology, and activation of distinct cell death pathways. This change caused by APAP overdose is more prominent in male compared to female mice. Lower susceptibility of female mice to APAP injury, potentially due to improved detoxification or repair mechanisms and the potential role of estradiol, has been reported previously [47,48]. Our study displayed higher levels of the non-toxic APAP-sulfate, APAP-GSH, and APAP itself in WT and GKO female mice. Analysis of early time points showed higher levels of these metabolites in WT female mice, indicating faster recovery of GSH in females, as noted previously by others [47]. This would indicate effective conjugation and clearance of intact APAP in females compared to that in males being partly responsible for the resistance of females to APAP hepatotoxicity.

Glo-1, a cytosolic enzyme, is part of the glyoxalase system, responsible for the detoxification of MEG, which provides protection against dicarbonyl stress [49]. Stress induced by APAP overdose further augmented the effects of Glo-1 deletion and significantly aggravated the levels of AGEs in GKO mice when compared to the corresponding WT mice. High concentrations of AGEs are known to activate RAGE, which is promoted by oxidative stress and inflammation, and have consequences in various liver disorders [50]. Indeed, the expression of RAGE was more pronounced in the GKO-APAP group compared to the respective saline-treated controls. The interaction of AGE and RAGE is expected to contribute to the propagation and acceleration of APAP-induced toxic effects. APAP overdose, however, did not lead to MGH1 accumulation and RAGE induction in wild-type mice, raising a question about the relevance of findings from GKO in a real-life scenario. The activation of compensatory mechanisms in compromised Glo-1 expression was observed in this study (Figure 5) and has been reported by others, which could explain the disconnect. Although the deletion of Glo-1 partly conveys the mechanism, additional studies on the effect of co-morbidities and dose-response studies with APAP could help describe the relevance of these findings in a clinical setting.

Consequently, hallmark oxidative stress markers, such as lipid peroxidation, protein carbonyl, and oxidized GSH levels, were elevated in GKO-APAP mice. High oxidative stress in GKO mice limited the efficient detoxification of APAP in GKO male mice, as seen by reduced levels of non-toxic APAP adducts with GSH and Cys, as well as sulfated APAP metabolites. Treatment with the Glo-1 cofactor ψ-GSH successfully mitigated ALT elevation and the biochemical consequences of APAP overdose irrespective of the Glo-1 status. Compromised Glo-1 expression in APAP-treated WT mice, compensatory mechanisms in GKO mice, and the Glo-1-independent antioxidant capability of ψ-GSH could account for the lack of Glo-1 dependence for ψ-GSH’s pharmacological action. Histopathological examination revealed striking changes in liver lipid accumulation in untreated and APAP-treated GKO livers, with hepatocyte degeneration caused by microvesicular steatosis. We analyzed the expression of ADFP to confirm microvesicular steatosis, which plays a key role in lipid homeostasis. In fact, ADFP knockout in mice attenuates hepatic steatosis [51]. Results of immunohistochemical and Western blot analysis showed the exacerbation of APAP-induced steatosis by Glo-1 deletion, while treatment with ψ-GSH suppressed this phenotype. Increased MEG levels due to Glo-1 deletion have been previously shown to elevate fatty acid synthase activity and resultant lipid accumulation in *Drosophila melanogaster* [52] and zebrafish [53] models. We biochemically confirmed the increased lipid content in APAP-treated animals using sulfo-phospho-vanillin assay, which is widely used for the measurement of total lipids in biological samples [17]. High-dose APAP caused increased liver lipid levels in both WT and GKO mice, with the highest levels found in the GKO-APAP group. The inability of ψ-GSH to completely reverse the lipid increase in this assay could be due to non-specific measurement of lipids, potentially including membrane lipids. This observation suggests a distinct degenerative pathology induced by Glo-1 deletion, which further intensifies APAP-induced liver toxicity. Increased AGE content with RAGE stimulation has also been found in liver steatosis and fibrosis in SD rats [50] and in NAFLD patients. It also induces inflammation, proliferation, and fibrosis in hepatic stellate cells (HSCs) by stimulating TGF-β1 expression [54]. The absence of functional Glo-1 also elevated necrosis, specifically necroptosis-induced cell death and diminished signs of apoptosis, in this study, as evident by the increased HMGB1 and RIPK3 levels and the absence of caspase-3 processing in APAP-treated GKO mice. The time course of APAP toxicity in these mice could help delineate the contribution of Glo-1 in initiating various cell death cascades. Differences in WT and GKO mice support the contribution of Glo-1 to necrosis, since its deletion shows the activation of programmed necrosis (necroptosis). Liver steatosis in the GKO-APAP group could trigger lethal lipotoxic signals, initiating hepatocyte degeneration, mitochondrial dysfunction, and fibrotic reactions, leading to NAFLD-type pathology [55], enhancing hepatotoxic effects of APAP. High-dose APAP itself causes the downregulation of Glo-1 expression (Figure 6A,B). It is plausible that depending on the magnitude and duration of APAP exposure, the APAP insult in wild-type mice may begin to mirror the effect of the loss of Glo-1 function, drawing a mechanistic interconnection between the disparate cell death pathways observed in WT and GKO mice.

The clinical antidote, NAC, effectively addresses the deleterious APAP effects by correcting the tissue redox status and quenching the reactive metabolite of APAP. However, its use is hampered by a narrow treatment window, gastrointestinal side effects, and anaphylaxis. Other means of alleviating oxidative stress could lead to therapeutic approaches to mitigate the hepatotoxicity of APAP poisoning. Exogenous administration and overexpression of antioxidant enzymes, SOD, and glutathione peroxidase have proved promising in mitigating APAP toxicity in preclinical studies [56,57]. Blockage of RAGE also leads to the attenuation of APAP-induced liver damage [6]. Diabetes/hyperglycemia, known to activate AGE/RAGE signaling, is considered one of the risk factors for acute liver injury [58]. Thus, regulating the consequences of toxic glucose metabolites, such as AGE formation, by balancing the cellular redox potential and inhibition of RAGE, rather than Glo-1 function restoration, would be expected to be an effective therapeutic strategy. Supplementation with bioavailable GSH analogs or precursors has shown promise in preclinical and clinical setting [13,59]. Indeed, ψ-GSH treatment showed benefits over NAC in our previous study related to APAP hepatotoxicity [13]. In addition to their innate antioxidant potential, such compounds are expected to engage GSH-dependent enzymes in restoring redox homeostasis. The results of this study highlight the importance of one such GSH enzyme, Glo-1, in modulating the hepatotoxic effects of APAP. Furthermore, the characterization of cell death mechanisms underscores the regulation of RIPK3-dependent mechanisms for the alleviation of APAP-induced liver injury. While this study demonstrates the development of steatosis in the livers of GKO mice, the precise role of the Glo-1 pathway in steatosis pathogenesis remains incompletely understood and warrants further investigation. While the resistance of female mice to APAP toxicity has been studied previously, such investigations in the context of the Glo-1 pathway could provide mechanistic insights. Additional studies are also needed to understand the time- and dose-dependent effects of APAP overdose on Glo-1 pathway intermediates and cell death mechanisms.

## 5. Conclusions

Taken together, our data demonstrate the important function of Glo-1 in liver homeostasis and in modulating glycation-induced oxidative stress resulting from acute toxic insults. Supplementation with an antioxidant GSH mimetic alleviates the lethal lipotoxic phenotype displayed in the liver tissue of Glo-1-deleted mice and prevents oxidative stress and hepatocyte degeneration. Such intervention could have application in the treatment of acute liver toxicity, such as that induced by extraneous agents or chronic liver illness leading to NAFLD or NASH pathology. Efforts to understand the time course of liver toxicity progression after APAP overdose and the factors responsible for the resistance of female mice to APAP toxicity are currently underway. This study adds greatly to our understanding of the Glo-1/AGE/RAGE axis in APAP-induced hepatotoxicity and emphasizes the importance of mitigating the redox imbalance to effectively address the initial stages of acute liver injury.

## Data Availability

The data presented in this study, such as raw data or original images, are contained in the article and Supplementary Materials. The chemical compound is available upon request from the corresponding author.

## Supplementary Materials

The following supporting information can be downloaded at https://www.mdpi.com/article/10.3390/antiox13060648/s1, Figure S1: Schematic of the role of glyoxalase 1 in the regulation of oxidative stress; Figure S2: Characterization of GKO mice; Figure S3: Deletion of Glo-1 reduces anxiety-like behavior; Figure S4: Analysis of serum creatinine, cholesterol and triglyceride levels in APAP-treated GKO mice; Figure S5: Analysis of serum APAP metabolites during early stages of hepatotoxicity; Figure S6: Effect of Glo-1 deletion on the levels of APAP metabolites and CYP2E1 activity; Figure S7: Liver catalase activity in mice treated with APAP; Figure S8: Quantification of liver AGE content by ELISA; Table S1: Concentrations of APAP metabolites in serum samples.
