# Supplementary material for: Deletion of Glyoxalase 1 Exacerbates Acetaminophen-Induced Hepatotoxicity in Mice
Source: Antioxidants (Basel). 2024 May 25;13(6):648. doi: 10.3390/antiox13060648 (PMC11200933; doi:10.3390/antiox13060648)
Supplement: Supplementary file 1 [file antioxidants-13-00648-s001.zip › antioxidants-2973113-supplementary.pdf]

Supplementary File 1

Supplementary Figures S1-S8 and Table S1

For

Deletion of Glyoxalase 1 exacerbates acetaminophen-induced hepatotoxicity in mice

Prakashkumar Dobariya[1,†], Wei Xie[1,†], Swetha Pavani Rao[1], Jiashu Xie[1], Davis M. Seelig[2,3], Robert Vince[1], Michael K. Lee[4,5], and Swati S. More[1,*]

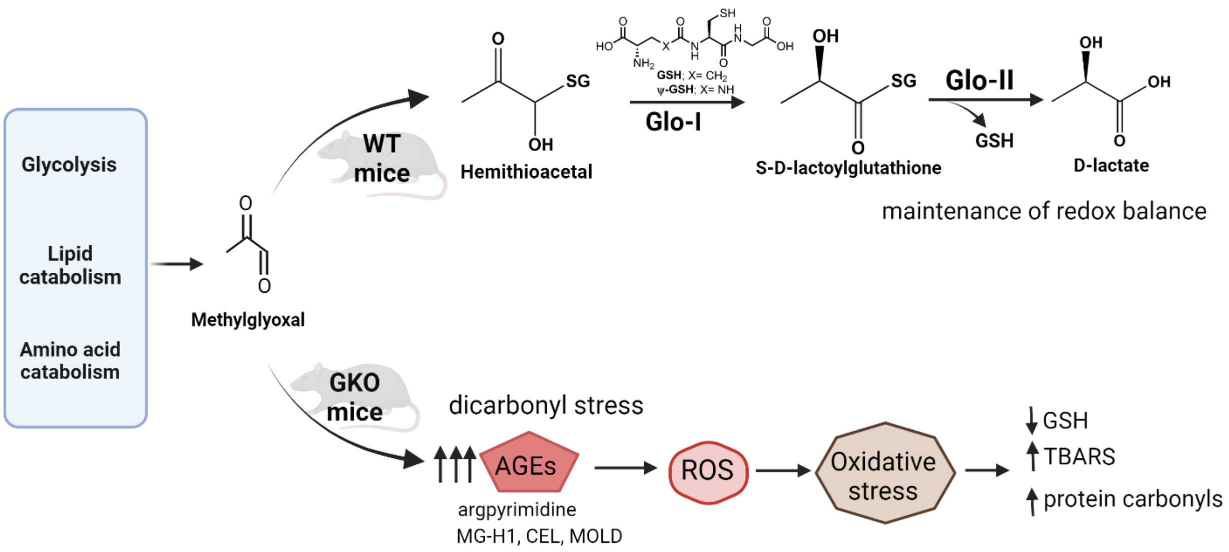

Figure S1. Role of glyoxalase 1 in the regulation of oxidative stress.

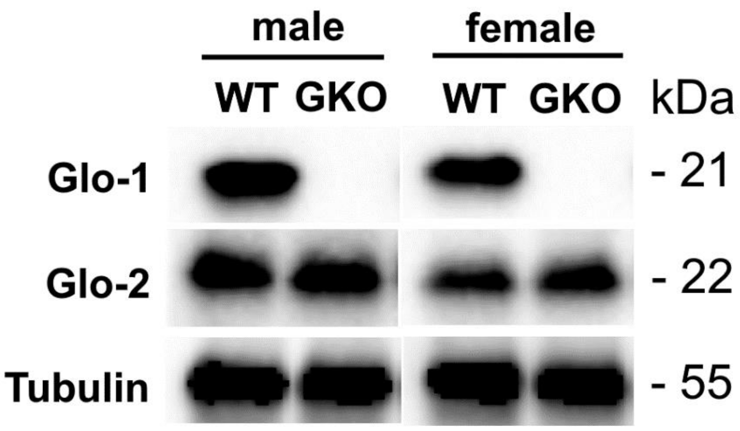

Figure S2. Characterization of GKO mice by Western blot for expression of Glo-1 and Glo-2. Deletion of Glo-1 was confirmed in GKO mice by the absence of protein band.

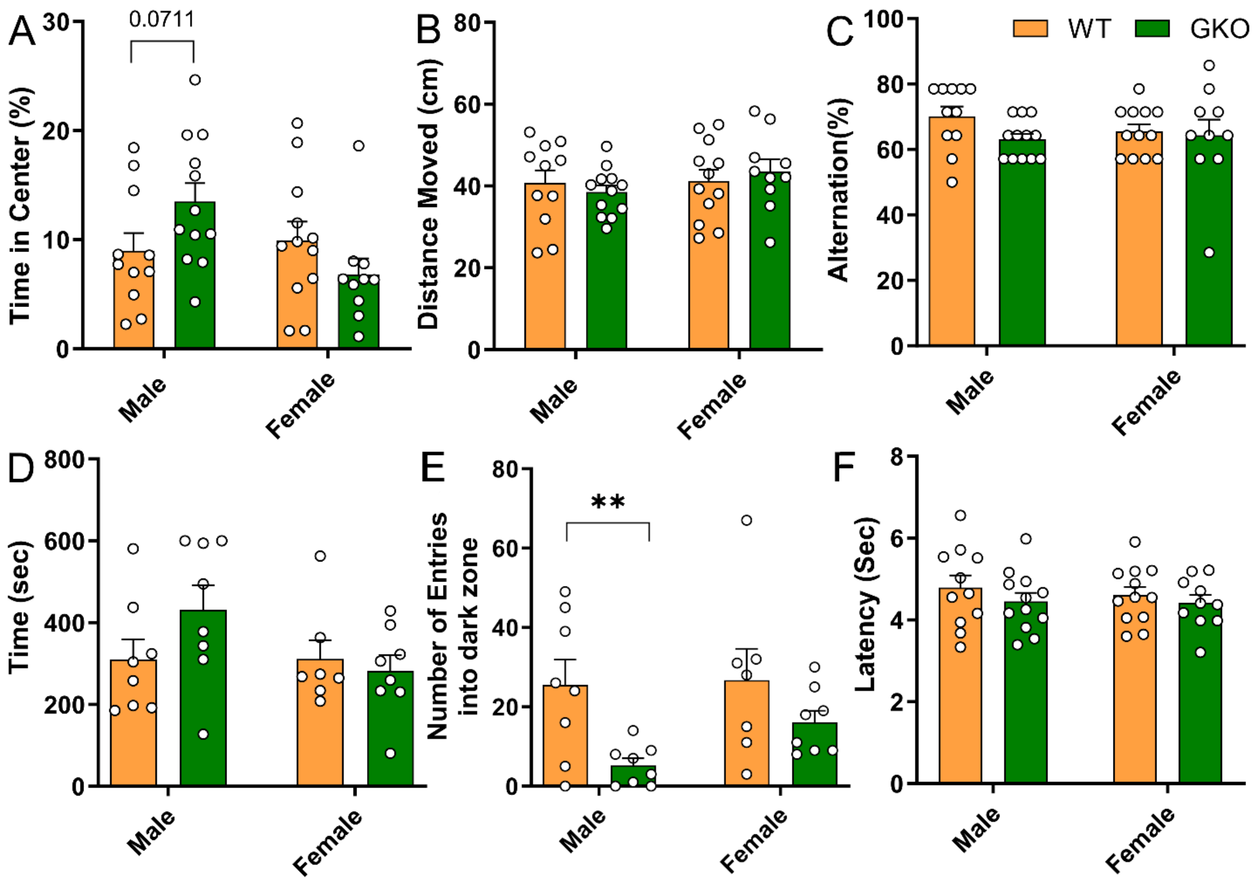

Figure S3. Deletion of Glo-1 reduces anxiety-like behavior. (A-B) Open field test showing time spent in the center zone (A) by age-matched WT and GKO mice; (B) total distance moved during the open filed test; (C) T-maze spontaneous alternation test showing %alternation rate; (D-E) Light-dark box test showing time spent in light zone (D) and number of transitions (E) during the testing period. (F) Tail flick test showing the latency of tail flick response. (n = 11-12 per group). Data are shown as mean ± SEM (* $p < 0.05$ by two-way ANOVA using Sidak multiple comparison post-hoc test).

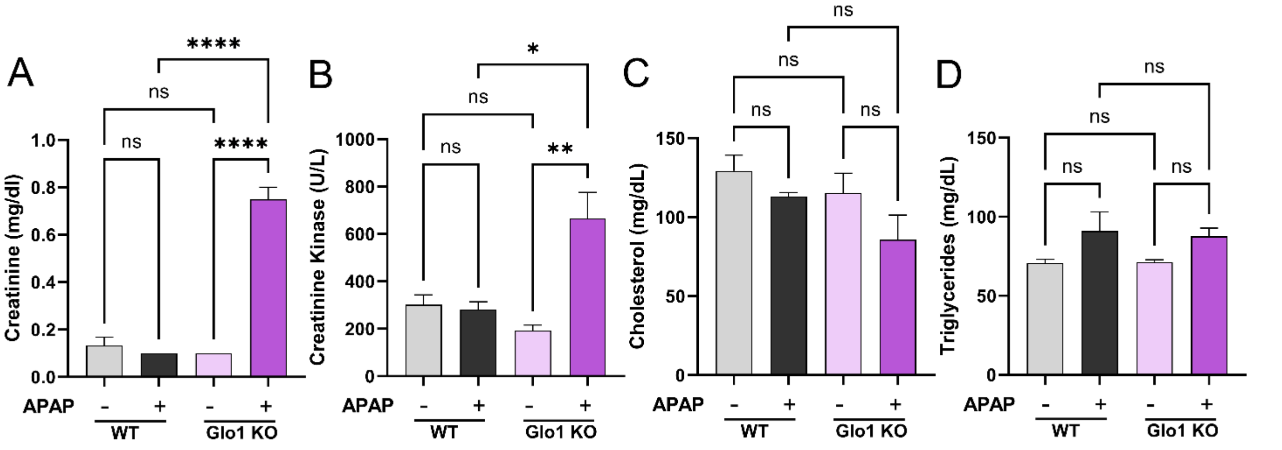

Figure S4. Analysis of serum creatinine (A), creatinine kinase (B), cholesterol (C) and triglyceride (D) levels of WT and GKO mice treated with high-dose APAP. Data are shown as mean ± SEM (*p < 0.05, ** p < 0.01, **** p < 0.0001 by one-way ANOVA using Sidak multiple comparison post-hoc test).

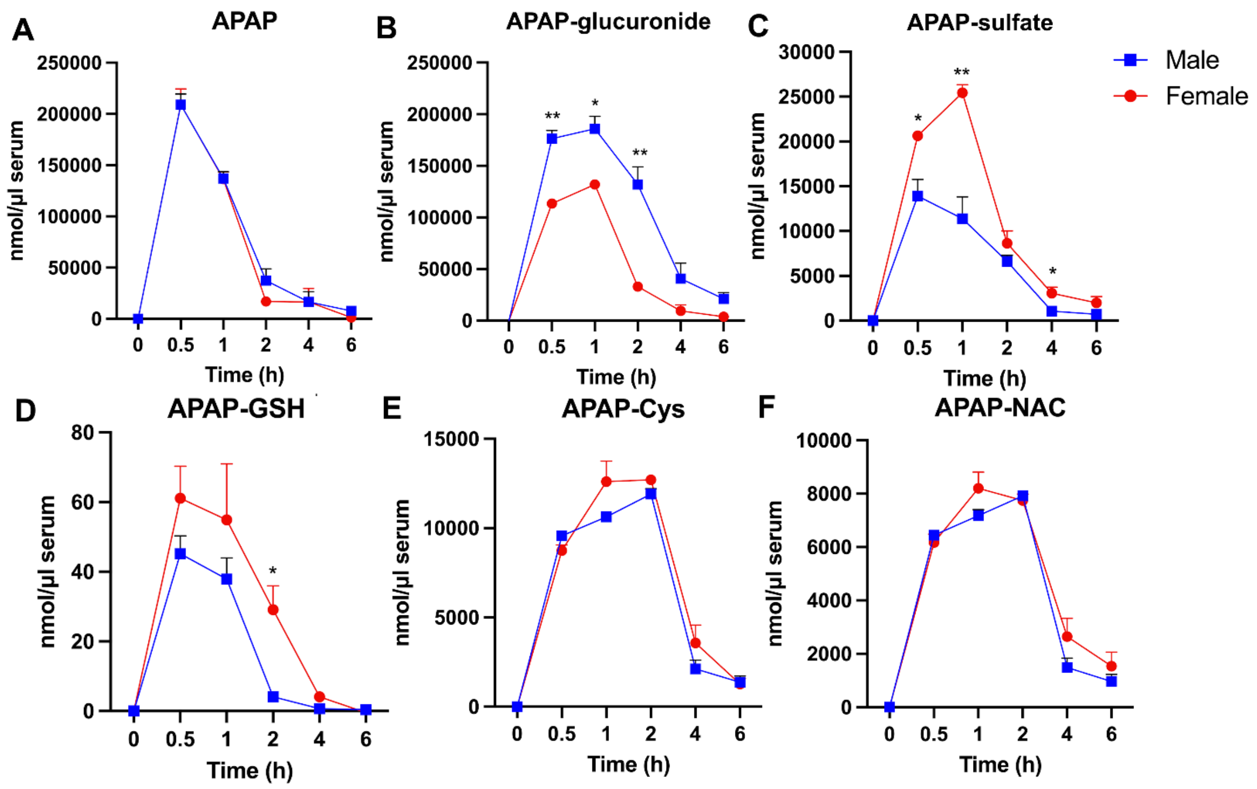

Figure S5. Analysis of serum APAP metabolites during early stages of hepatotoxicity. Serum samples from fasted wild-type C57BL/6 male and female mice collected at 0.5, 1, 2, 4 and 6 hours post APAP overdose were subjected to metabolite identification by LC-MS/MS. Concentrations of each APAP metabolite are displayed as mean ± SEM. (A) APAP, (B) APAP-glucuronide, (C) APAP-sulfate, (D) glutathione adduct (APAP-GSH), (E) cysteine adduct (APAP-Cys), (F) N-acetylcysteine adduct (APAP-NAC) (* p < 0.05; **, p < 0.01; unpaired Student t-test).

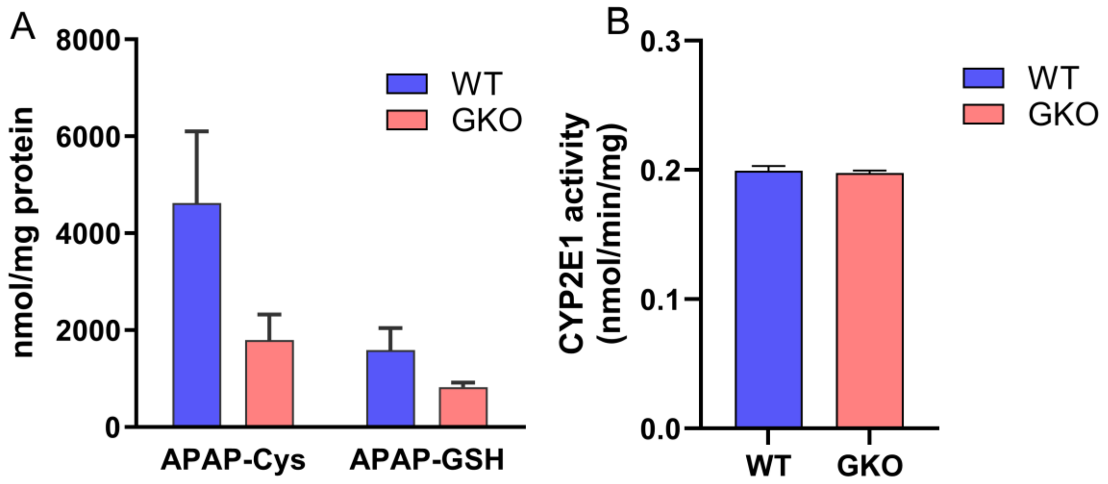

Figure S6. (A) Effect of Glo-1 deletion on the levels of APAP metabolites, APAP-Cys and APAP-GSH in liver tissues. Liver tissues were collected from fasted WT and GKO male mice 1 hour after APAP administration. The metabolite levels were measured by LC-MS/MS. (B) The baseline function of CYP2E1 was unchanged in the WT and GKO mouse livers.

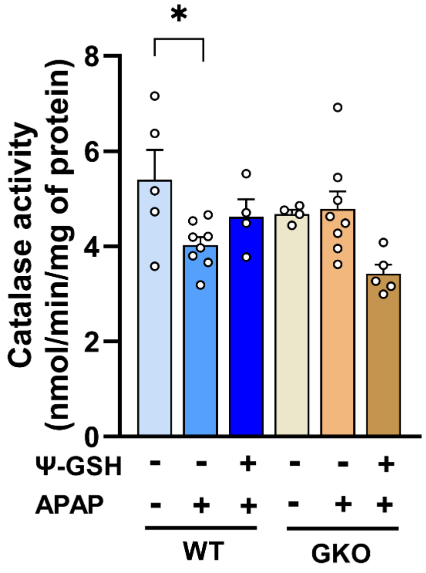

Figure S7. Liver catalase activity in mice treated with APAP. Except for the reduced catalase activity in WT-APAP group compared to WT-saline, no significant differences were noted within different treatment groups. Data are shown as mean ± SEM (* $p < 0.05$, one-way ANOVA followed by Tukey's multiple comparison post-hoc test).

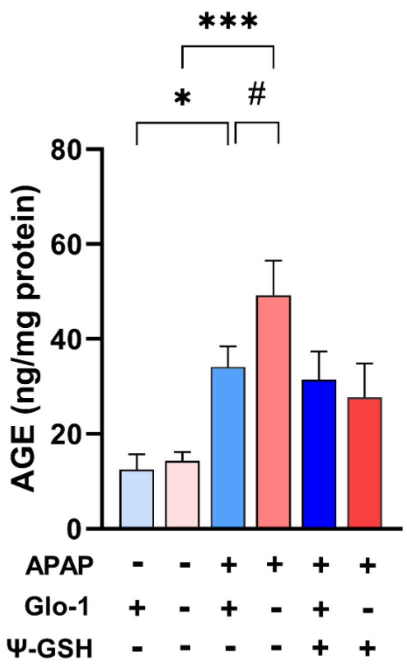

Figure S8. Quantification of liver AGE content by a commercial mouse AGE-ELISA. APAP treatment increased AGE content in WT and GKO livers. Ψ-GSH treatment was unable to reduce AGE levels. Data are shown as mean ± SEM (* $p < 0.05$, *** $p < 0.001$, one-way ANOVA followed by Tukey’s multiple comparison post-hoc test; # $p = 0.05$, *t*-test)

Table S1. Concentrations of APAP metabolites in serum samples expressed as nmol per μL serum

Metabolite	Wild Type	GKO	Wild Type	GKO
	Male		Female	
APAP	690.88 ± 147.57	409.45 ± 132.56	1476.31 ± 538.24	2370.11 ± 565.77 (0.0150* *vs* GKO male)
APAP-glucuronide	1415.64 ± 678.44	671.09 ± 161.73	3019.23 ± 1586.09	7347 ± 1880.66 (0.0459* *vs* WT male; 0.0168 *vs* GKO male)
APAP-sulfate	230.12 ± 41.26	65.99 ± 36.01 (0.0241* *vs* WT male)	1987.66 ± 338.46 (0.0021** *vs* WT male)	2343.81 ± 330.71 (0.0005*** *vs* GKO male)
APAP-GSH	28.08 ± 8.25	6.147 ± 3.03 (0.0187* *vs* WT male)	34.48 ± 22.47	41.15 ± 4.17 (0.0001*** *vs* GKO male)
APAP-Cys	916.57 ± 338.22	150.19 ± 32.66 (0.065 *vs* WT male)	561.63 ± 251.19	352.31 ± 118.89
APAP-NAC	427.30 ± 73.82	88.28 ± 24.67 (0.0048** *vs* WT male)	637.24 ± 347.69	250.43 ± 79.79
APAP-OMe	1.96 ± 1.08	0.19 ± 0.22 (0.0184* *vs* WT male)	2.66 ± 1.11	4.01 ± 1.58 (0.0030** *vs* GKO male)
